# Stationary relativistic jets

**DOI:** 10.1186/s40668-015-0013-y

**Published:** 2015-11-05

**Authors:** Serguei S Komissarov, Oliver Porth, Maxim Lyutikov

**Affiliations:** 1 School of Mathematics, University of Leeds, Leeds, LS29JT UK; 2 Department of Physics and Astronomy, Purdue University, West Lafayette, 47907-2036 USA; 3 Centre for mathematical Plasma Astrophysics, Department of Mathematics, KU Leuven, Celestijnenlaan 200B, Leuven, 3001 Belgium

**Keywords:** jets, relativity, magnetic fields, hydrodynamics, numerical methods

## Abstract

In this paper we describe a simple numerical approach which allows to study the structure of steady-state axisymmetric relativistic jets using one-dimensional time-dependent simulations. It is based on the fact that for narrow jets with $v_{z}\approx c$ the steady-state equations of relativistic magnetohydrodynamics can be accurately approximated by the one-dimensional time-dependent equations after the substitution $z=ct$. Since only the time-dependent codes are now publicly available this is a valuable and efficient alternative to the development of a high-specialised code for the time-independent equations. The approach is also much cheaper and more robust compared to the relaxation method. We tested this technique against numerical and analytical solutions found in literature as well as solutions we obtained using the relaxation method and found it sufficiently accurate. In the process, we discovered the reason for the failure of the self-similar analytical model of the jet reconfinement in relatively flat atmospheres and elucidated the nature of radial oscillations of steady-state jets.

## Introduction

Highly collimated flows of plasma from compact objects of stellar mass, like young stars, neutron stars and black holes, as well as supermassive black holes residing in the centers of active galaxies is a wide-spread phenomenon which has been and will remain the focal point of many research programs, both observational and theoretical. Some features of these cosmic jets, like moving knots, are best described using time-dependent fluid models. However, most of these jets have sufficiently regular global structure, which is indicative of steady production and propagation and promotes development of stationary models. Such models are also easier to analyze, and they are very helpful in our attempts to figure out the key factors of the jet physics.

The simplest approach to steady-state flows is to completely ignore the variation of flow parameters across the jet. This allows to reduce the complicated system of non-linear partial differential equations (PDEs) describing the jet dynamics to a set of ordinary differential equations (ODEs) which can be integrated more easily (*e.g.* Blandford and Rees 1974; Komissarov 1994). A similar reduction in the dimensionality is achieved in self-similar models, where unknown functions depend only on a combination of independent variables known as a self-similar variable. This also allows to reduce the original PDEs to a set of ODEs (*e.g.* Blandford and Payne 1982; Vlahakis and Tsinganos 1998). While providing important test cases and useful insights, this approach is not sufficiently robust - boundary and other conditions that select such exceptional solutions are not always present in nature.

**Figure 1:**
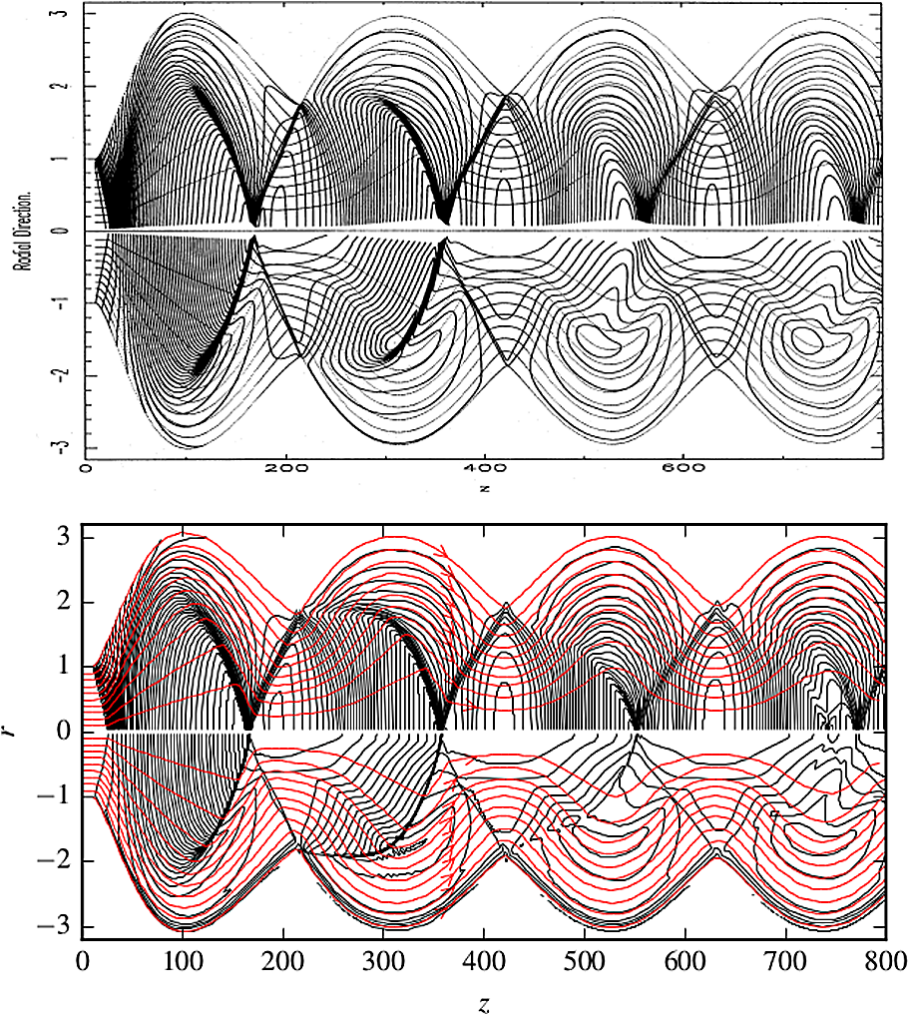
**Reconfinement of the** $\pmb{M_{\mathrm{j}}=15}$ **,** $\pmb{T_{\mathrm{j}}=\sqrt{10}\times10^{13}\mathrm{K}}$ **jet.** The top panel is a reproduction of Figure 3 from B94. The bottom panel shows the solution obtained with our method. In each panel, the top halves show 50 pressure contours (spaced by the factor of 1.18) and the bottom halves show the temperature parameter $\tau\equiv\rho h/(\rho h - p)$ in 50 contours (spaced by the factor of 1.003). The light gray lines are streamlines.

As it is well known to engineers working on aircraft jet engines, supersonic jets naturally develop quasi-periodic stationary chains of internal shocks, similar to what is shown in Figure 1. These shocks emerge as a part of the adjustment of the jet pressure to that of the surrounding air. Interestingly, bright knots are often seen in cosmic jets and they are often interpreted as shocks (*e.g.* Falle and Wilson 1985; Daly and Marscher 1988; Gómez and Marscher 2000; Arshakian et al. 2010; Walker 1997). Some of these knots are known to be traveling and they must be part of the jet’s non-stationary dynamics. Others appear to be static and hence connected to the underlying quasi-steady-state structure of these cosmic jets. Quite often, the knots form quasi-periodic chains, reminiscent of those seen in aerodynamic jets. If the similarity is not accidental, then these knots are also related to the process of pressure adjustment. In particular, we expect the powerful cosmic jets to be expanding freely soon after leaving their central engines and to become confined by external pressure again only much later (*e.g.* Daly and Marscher 1988; Komissarov and Falle 1997). The first shock driven into the jet by the external pressure is called the reconfinement shock. Given the growing observational evidence of stationary knots in cosmic jets, there has been a increase of interest to the reconfinement process among theorists in recent years (*e.g.* Nalewajko and Sikora 2009; Nalewajko 2012; Bromberg and Levinson 2007; Bromberg and Levinson 2009; Kohler et al. 2012; Kohler and Begelman 2012; Kohler and Begelman 2015). One of the key aims of these studies was to come up with approximate analytical or semi-analytical solutions for the structure of steady-state jets.

Obviously, such shocked flows cannot be described by one-dimensional (1D) and self-similar models, which we mentioned earlier, and more complex, at least two-dimensional (2D), models have to be applied instead. The system of steady-state equations of compressible fluid dynamics, not to mention magnetohydrodynamics, is already very complicated and generally requires numerical treatment. One of the ways of finding its solutions involves integration of the original time-dependent equations in anticipation that if the boundary conditions are time-independent then the time-dependent numerical solution will naturally evolve towards a steady-state (*e.g.* Ustyugova et al. 1999; Komissarov et al. 2009; Tchekhovskoy et al. 2008). One clear advantage of this approach is that it allows to use standard codes for time-dependent fluid dynamics. Such codes are now well advanced and widely available. However, this type of the relaxation approach is characterized by slow convergence and hence rather expensive.

In order to speed up the convergence, one can use other relaxation methods, which are developed specifically for integrating steady-state equations (*e.g.* May and Jameson 2011). They often involve a relaxation variable which is called ‘pseudo time’. However, this time evolution is not realistic but designed to drive solutions towards a steady-state in the fastest way possible. The only disadvantage of this approach is that it involves development of a specialised computer code dedicated to solving only steady-state problems. The authors are not aware of such codes for relativistic hydro- and magnetohydrodynamics.

For supersonic flows, the system of steady-state equations turns out to be hyperbolic, with one of spatial coordinates playing the role of time (Glaz and Wardlaw 1985). (In the case of magnetic jets, the speed of sound is replaced with the fast magneto-sonic speed and we classify flows as sub-, tran-, or super-sonic based on its value compared to the flow speed.) In this case, one can find steady-state solutions utilising numerical methods which were designed specifically for hyperbolic systems, like the method of characteristics or ‘marching’ schemes. These methods have been used in the past in applications to relativistic jets (*e.g.* Daly and Marscher 1988; Wilson and Falle 1985; Wilson 1987; Bowman 1994; Bowman et al. 1996) but publicly available codes do not exist yet. Their development is as time-consuming as that of time-dependent codes whereas the range of applications is much more limited. This explains their current unavailability. Moreover, when flow becomes subsonic, even very locally, this approach fails.

In this paper, we propose a new approach, which allows to find approximate numerical steady-state jet solutions rather cheaply and using widely available computer codes. To be more precise, we focus on highly relativistic narrow axisymmetric jets and show that in this regime the 2D steady-state equations of Special Relativistic MHD (SRMHD) are well approximated by 1D time-dependent equations of SRMHD. Like in the standard marching schemes, the spatial coordinate along the jet plays the role of time. This allows us to find steady-state structure of axisymmetric jets by carrying out basic 1D SRMHD simulations, which can be done with very high resolution even on a very basic personal computer. In such simulations, no special effort is needed to preserve the magnetic field divergence-free and the computational errors associated with multi-dimensionality are eliminated. As the result, more extreme conditions can be tackled. Here we focus only on relativistic jets, because of our interest to AGN and GRB jets, but we see no reason why this approach cannot be applied to non-relativistic hypersonic jets as well. Our approach is closely related to the so-called ‘frozen pulse’ approximation, which also utilizes the similarity between the steady-state and time-dependent equations describing ultra-relativistic flows (Piran et al. 1993; Vlahakis and Königl 2003; Sapountzis and Vlahakis 2013). In this approximation, the steady-state equations are used to analyze the dynamics of time-dependent flows. The similarity between 1D time-dependent models and 2D steady-state jet solutions has been noted before, in particular in Matsumoto et al. (2012).

In order to study the potential of this new approach we have carried out a number of test simulations and compared the results obtained in this way with both analytical models and numerical solutions obtained with more traditional methods. The results are very encouraging and allow us to conclude that this method is viable and can be used in a wide range of astrophysical applications.

## Approximation

We start by writing down the time-dependent equations of Special Relativistic Magnetohydrodynamics (SRMHD). In this section we use units where the speed of light $c=1$ and the factor $1/4\pi$ does not appear in the expression for the electromagnetic energy density. The components of vectors and tensors are given in normalized bases. The evolution equations of SRMHD include the continuity equation

1 $$ \partial _{t} {\rho\Gamma} + \mathbf {\nabla}\cdot(\rho\Gamma \mathbf {v})=0 , $$

the Faraday equation

2 $$ \partial _{t} \mathbf {B}+ \mathbf {\nabla } \times \mathbf {E} = 0 $$

and the energy-momentum equation

3 $$ \partial _{t} {T^{t\mu}}+\nabla_{j}{T^{j\mu}}=0 , $$

where

4 $$ T^{\nu\mu}=T^{\nu\mu} _{\mathrm {hd}} + T^{\nu\mu} _{\mathrm {em}} $$

is the total stress-energy-momentum tensor,

5 $$ T^{\nu\mu} _{\mathrm {hd}} = wu^{\nu}u^{\mu}+pg^{\nu\mu} $$

is stress-energy-momentum tensor of matter and the components of the electromagnetic stress-energy-momentum tensor are

6 $$\begin{aligned}& T^{tt}_{\mathrm {em}} =\bigl(E^{2}+B^{2}\bigr)/2 , \end{aligned}$$

7 $$\begin{aligned}& T^{ti}_{\mathrm {em}} =( \mathbf {E} \times \mathbf {B})^{i} , \end{aligned}$$

8 $$\begin{aligned}& T^{ij}_{\mathrm {em}} =-\bigl(E^{i}E^{j}+B^{i}B^{j} \bigr)+\frac{1}{2}\bigl(E^{2}+B^{2}\bigr)g^{ij} . \end{aligned}$$

In these equations, **B** and **E** are the vectors of magnetic and electric fields respectively, *p*, *ρ* and *w* are the thermodynamic pressure, rest-mass density of matter and relativistic enthalpy of matter respectively, **v** is the velocity vector, Γ is the Lorentz factor and **g** is the metric tensor of space. These equations are to be supplemented with Equation of State $w=w(\rho,p)$ and the Ohm’s law of ideal MHD

9 $$ \mathbf {E}= - \mathbf {v} \times \mathbf {B} . $$

Finally, the magnetic field is divergence-free

10 $$ \mathbf {\nabla } \cdot \mathbf {B}=0 . $$

In this analysis, we focus on axisymmetric jets and adopt a cylindrical coordinate system with the *z* axis coincident with the jet symmetry axis. We consider only narrow jets, so that

11 $$ \frac{r}{z} \ll1 . $$

We also constrain ourselves with a relatively simple magnetic configurations where the divergence-free condition leads to

12 $$ \frac{B^{r}}{B^{z}} \simeq\frac{r}{z} \ll1 . $$

In axisymmetry, the steady-state Faraday equation implies $E^{\phi}=0$. When combined with Eq. (9), this result yields

13 $$ \frac{v^{r}}{v^{z}} = \frac{B^{r}}{B^{z}} \ll1 . $$

Thus, the radial components of both the magnetic field and the velocity vectors are small compared to their axial components.

We also assume that $v^{\phi}\ll1$. In fact, in the case of magnetically accelerated jets,

$$v^{\phi}\simeq(r_{\mathrm {lc}}/r) $$

when $r\gg r_{\mathrm {lc}}$, the radius of light cylinder (see Eq. (66) in Komissarov et al. (2009)). Thus, this is a good approximation for astrophysical jets. For a highly relativistic flow, the condition $v^{z} \gg v^{r},v^{\phi}$ means

14 $$ v^{z} \simeq1 . $$

Following the standard flux freezing argument, along the jet $B^{\phi}/B^{z} \simeq(r_{\mathrm {j}}/r_{\mathrm {lc}})^{-1}$, where and $r_{\mathrm {j}}$ is the jet radius (This argument does not apply to turbulent jets, which are non-axisymmetric and allow non-trivial conversion of components.). Hence one may argue that far away from the central engine

15 $$ B^{\phi}\gg B^{z} . $$

In order to introduce the key idea of our approach we consider first the steady-state continuity equation:

16 $$ \partial _{z} \bigl(\rho\Gamma v^{z}\bigr) + \nabla _{r} \bigl(\rho\Gamma v^{r}\bigr)=0 . $$

Using the condition (14) we may replace $v^{z}$ with unity. This makes Eq. (16) identical to the 1D time-dependent version of the continuity equation. In order to stress this point we replace *z* with *t* and write:

17 $$ \partial _{t} (\rho\Gamma) + \nabla _{r} \bigl(\rho\Gamma v^{r}\bigr)=0 . $$

Similarly, all 2D steady-state equations can be approximated by the corresponding 1D time-dependent equations.

Let us show this for the equations of magnetic field. The 1D version of the divergence free condition reads

$$\partial _{r} \bigl(rB^{r}\bigr) = 0 \quad\mbox{or}\quad r B^{r} = \mbox{const}. $$

Thus if $B^{r}$ vanishes outside of the jet, which is expected when it is in direct contact with ISM, then one has to put $B^{r}=0$ everywhere in the 1D model. As we shell see, the terms involving $B^{r}$ are sub-dominant in all other equations and hence this is a reasonable simplification. Moreover, once the 1D solution is found, one can substitute the determined $B^{z}(r,z)$ into the 2D divergence free condition and solve it for $B^{r}(r,z)$. The result can then be used to verify that $B^{r}(r,z) \ll B^{z}(r,z)$.

The *ϕ* component of the Faraday equation can be written as

18 $$ \partial _{t}B^{\phi}-rB^{i}\partial _{i} \biggl(\frac{v^{\phi}}{r} \biggr) + \partial _{i} \bigl(v^{i} B^{\phi}\bigr) =0 , $$

where $i={r,z}$. In steady-state, the first term vanishes, the next two terms are of the order $B^{z} v^{\phi}/z$ and small compared to the last two terms, which are of the order $B^{\phi}v^{z}/z$. Removing these small terms we obtain the approximate steady-state equation

19 $$ \partial _{z} \bigl(v^{z} B^{\phi}\bigr) + \partial _{r} \bigl(v^{r} B^{\phi}\bigr) =0 . $$

Finally, we replace $v^{z}$ with unity, *z* with *t*, and obtain

20 $$ \partial _{t} B^{\phi}+ \partial _{r} \bigl(v^{r} B^{\phi}\bigr) =0 . $$

This is indeed the 1D version of the *ϕ* component of Eq. (18). Now consider the *z* component of the Faraday equation,

21 $$ \partial _{t} B^{z} -B^{i}\partial _{i}v^{z} + \frac{1}{r} \partial _{i} \bigl(r v^{i} B^{z}\bigr) = 0 . $$

The last two terms of this equation are of the order $v^{z} B^{z}/z \simeq B^{z}/z$. On the other hand, the second and the third terms are much smaller because of the special status of $v^{z}$, which is approximately constant, and hence $B^{z}\partial _{z}v^{z} \ll B^{z} (v^{z}/z)$. Removing these small terms, we obtain the approximate steady-state equation

22 $$ \partial _{z} \bigl(v^{z} B^{z}\bigr) + \frac{1}{r} \partial _{r} \bigl(r v^{r} B^{z}\bigr) =0 . $$

Now once again we replace $v^{z}$ with unity and *z* with *t* to obtain

23 $$ \partial _{t} B^{z}+ \frac{1}{r} \partial _{r} \bigl(r v^{r} B^{z}\bigr) =0 , $$

which is the 1D version of the *z* component of Eq. (18).

Finally, we analyze the energy-momentum equations. These can be written as

24 $$ \partial _{t} {T^{t\mu}} + \partial _{z}T^{z\mu} + \nabla_{r}{T^{r\mu}}=0 , $$

so the steady-state versions are

25 $$ \partial _{z}T^{z\mu} + \nabla_{r}{T^{r\mu}}=0 . $$

These already have the same form as the 1D time-dependent equations, so we only need to show that

26 $$ T^{z\mu}\simeq T^{t\mu} . $$

Let us start with the hydrodynamic contribution. First, we notice that

$$\begin{aligned}& T^{tt}_{\mathrm {hd}} = w\Gamma^{2}-p \simeq w\Gamma^{2} \quad\mbox{as } \Gamma\gg 1 ; \\& T^{tz}_{\mathrm {hd}} = w\Gamma^{2} v^{z} \simeq w \Gamma^{2} \quad\mbox{as } v^{z}\simeq1 . \end{aligned}$$

Thus, $T^{zt}_{\mathrm {hd}}\simeq T^{tt}_{\mathrm {hd}}$. Then we notice that

$$\begin{aligned}& T^{ti}_{\mathrm {hd}} = w\Gamma^{2}v^{i} ; \\& T^{zr}_{\mathrm {hd}} = w\Gamma^{2} v^{z} v^{r} \simeq w\Gamma^{2} v^{r} ; \\& T^{zz}_{\mathrm {hd}} = w\Gamma^{2} v^{z} v^{z} \simeq w\Gamma^{2} v^{z} ; \end{aligned}$$

Thus, $T^{zi}_{\mathrm {hd}}\simeq T^{ti}_{\mathrm {hd}}$.

Now we inspect the electromagnetic contributions. First, we find good estimates for the components of electric field. From Eq. (9) it follows that

27 $$ E^{r} \simeq B^{\phi} $$

and

$$E^{z}=B^{r}v^{\phi}-B^{\phi}v^{r} \ll E^{r} . $$

In fact, it is easy to show that

28 $$ E^{z} \simeq-B^{\phi}v^{r} . $$

Indeed, for magnetically accelerated jets $B^{\phi}\simeq\Omega r B^{z}$ (*e.g.* Komissarov et al. 2009) for $r\gg r_{\mathrm {lc}}$. Hence

$$v^{r} B^{\phi}\simeq v^{r}\Omega r B^{z} \simeq(r/r_{\mathrm {lc}}) B^{r} \gg B^{r} \gg B^{r}v^{\phi}. $$

Using these estimates we find that

$$\begin{aligned}& T^{tt}_{\mathrm {em}} = \frac{1}{2}\bigl(E^{2} + B^{2}\bigr) \simeq B_{\phi}^{2} ; \\& T^{tz}_{\mathrm {em}} = ( \mathbf {E} \times \mathbf {B})^{z} \simeq E^{r} B^{\phi}\simeq B_{\phi}^{2} , \end{aligned}$$

and hence $T^{zt}_{\mathrm {em}}\simeq T^{tt}_{\mathrm {em}}$. Moreover,

$$T^{zz}_{\mathrm {em}} = -\bigl(E_{z}^{2}+B_{z}^{2} \bigr) +\frac{E^{2}+B^{2}}{2} \simeq B_{\phi}^{2} , $$

and hence $T^{zz}_{\mathrm {em}}\simeq T^{tz}_{\mathrm {em}}$ as well. Next we show that $T^{z\phi} _{\mathrm {em}}\simeq T^{t\phi} _{\mathrm {em}}$. Indeed,

$$T^{t\phi} _{\mathrm {em}} = E^{z}B^{r} - E^{r}B^{z} \simeq- E^{r}B^{z} , $$

and

$$T^{z\phi} _{\mathrm {em}} = -\bigl(E^{z} E^{\phi}+B^{z} B^{\phi}\bigr) \simeq-B^{z} E^{r} . $$

Finally, we show that $T^{zr}_{\mathrm {em}}\simeq T^{tr}_{\mathrm {em}}$. First, we find straight away that

$$T^{tr}_{\mathrm {em}} = -E^{z} B^{\phi}\quad\mbox{and}\quad T^{zr}_{\mathrm {em}} = -\bigl(E^{z}E^{r}+B^{z}B^{r} \bigr) . $$

Since $E^{z}E^{r} \simeq E^{z} B^{\phi}$, we only need to show that $B^{z}B^{r}$ is significantly smaller compared to these terms. This is indeed the case as $B^{z}B^{r} \simeq v^{r} B_{z}^{2}$ whereas using Eqs. (27) and (28) we obtain $E^{z}E^{r} \simeq v_{r} B_{\phi}^{2} \gg v^{r} B_{z}^{2}$.

Thus, within our approximation the steady-state 2D equation of energy-momentum reduces to

29 $$ \partial _{t} {T^{t\mu}} + \nabla_{r}{T^{r\mu}}=0 , $$

which is the 1D time-dependent energy-momentum equation.

Given that in relativistic fluid dynamics small differences between the magnitudes of energy and momentum may result in huge variations of Lorentz factor and even lead to inconsistency, one could feel uneasy about the approximations we make. However, the final result is *exactly* the system of 1D time-dependent SRMHD and this means that self-consistency is not compromised. For example, the flow speed will not exceed the speed of light because of the errors of our approximation.

Our approach is similar to ‘marching’ - we compute solution for a downstream jet cross-section using only the previously found solutions for upstream cross-sections. Strictly speaking, this requires the flow to be super-sonic for unmagnetized jets and super-fast-magnetosonic for magnetized ones (Wilson and Falle 1985; Dubal and Pantano 1993). However, in our derivations we never had to utilize this condition. This suggests that it is not required when we wish to find only approximate solutions. For example, one may argue that the fact that information can propagate upstream does not necessarily imply that this always has a strong effect on the flow - the upstream-propagating waves could be rather weak. If so, we may still apply our method to jets where the supersonic condition is not fully satisfied, but we always need to check that the conditions (11)-(15) of our approximation hold for obtained solutions.

## Numerical implementation

The analysis of Section 2 shows that as long as they are applied to narrow jets with high Lorentz factor, the axisymmetric steady-state equations of SRMHD are very close to 1D time-dependent equations of SRMHD in cylindrical geometry. This suggests that it may be possible to use time-dependent simulations with 1D SRMHD codes to study the 2D structure of steady-state jet solutions. However in order to be able to do this, we also need to find a way of accommodating the 2D boundary conditions of steady-state problems in such simulations.

For 2D supersonic flows we need to fix all flow parameters at the jet inlet and impose some conditions at the jet boundary, consistent with it being a stationary contact wave. No boundary conditions are needed for the outlet boundary - its flow parameters are part of the solution. In the corresponding 1D problem, the 2D boundary conditions at the inlet boundary simply become the initial conditions of the 1D Cauchy problem. The final 1D solution corresponds to the slice of the 2D solution at the outlet boundary. As to the contact discontinuity at the 2D jet boundary, the situation is not that trivial.

Suppose that the total pressure at this boundary is a function of *z*, $p=p_{\mathrm {b}}(z)$. When we replace *z* with *t* this becomes $p=p_{b}(t)$. Thus we need somehow to impose time-dependent boundary conditions. In the simulations presented below, the following approach was utilised: (1) we extend the computational domain so that it includes the external gas, (2) we track the point separating the jet from the external gas and (3) we reset the external gas parameters according to the prescribed functions of time every computational time step.

In order to locate the boundary separating the jet from the external gas, we employ a simplified version of the level-set method (Osher and Sethian 1988; Sethian and Smereka 2003). Namely, we introduce the passive scalar *τ*, which satisfies the advection equation

30 $$ \partial _{t}(\Gamma\rho\tau) + \frac{1}{r} \partial _{r}\bigl(r\Gamma v^{r} \rho\tau \bigr) = 0 . $$

The initial solution has a smooth distribution of this scalar

31 $$ \tau=\frac{1}{2} \biggl(1 - \tanh\frac{r-r_{j}}{\Delta} \biggr) , $$

with the value $\tau=0.5$ corresponding to the jet boundary (In the test simulations, we used $\Delta=0.3r_{j}$.). During the simulations, the condition $\tau <0.5$ was used to identify the external gas.

After the reset, the 1D jet boundary is no longer a contact but a more general discontinuity. In particular, the jet plasma will generally have radial velocity component. If it is positive, but in the external gas it is set to zero, then a shock wave will launched into the jet when this discontinuity is resolved. If it is negative, then this will be a rarefaction wave. On the one hand, this reflects how the information about changing environment is communicated to the interior of a steady-state jet. On the other hand, in 1D simulations the strength of the emitted wave depends on the external density - higher density, and hence lower temperature, will result in stronger waves moving into the jet. This is obviously not so for 2D steady-state jets, which react only to the external pressure. Thus additional measures need to be undertaken. First, in order to negate the effect of the radial velocity jump at the jet boundary, the radial velocity of the external gas is reset not to zero but to its value at the last jet cell. Second, in order reduce the role of the external gas inertia, it helps to set its density to a low value, so that its sound speed becomes relativistic. Although we have not tried this, one could set the polytropic index of the external gas to $\Gamma=2$, which would make the sound speed of ultra-relativistically hot gas equal to the speed of light.

## Examples

### Bowman’s jet

To test the validity of our approach, we first use our method to reproduce the numerical steady-state solutions for supersonic unmagnetized jets obtained by Bowman (1994), B94, using the marching scheme described in Wilson (1987). In this study pressure-matched uniform jets with zero opening angle are injected into an atmosphere with the pressure distribution

32 $$ p(z) = p_{0} \biggl[ \biggl( \frac{z}{z_{\mathrm{s}}} \biggr)^{-2} + \biggl(1-\frac{z_{\mathrm{s}}}{z} \biggr) \biggl( \frac{z_{\mathrm{s}}}{z_{\mathrm{c}}} \biggr)^{2} \biggr] $$

with $z_{\mathrm{s}}=10$, $z_{\mathrm{c}}=50$. According to this equation, the external pressure initially decreases almost as fast as $\propto z^{-2}$ but at $z>z_{\mathrm {c}}$ becomes uniform. The initial jet radius $r_{0}=1$ and the injection nozzle is located at $z=z_{\mathrm {s}}$. The equation of state is that of Synge (1957) for an electron-proton plasma. The initial jet pressure $p_{\mathrm {j}}=p_{0}$. For the comparison we selected the model with the Mach number $M_{\mathrm {j}}=15$ and the initial temperature $T_{\mathrm {j}}=\sqrt{10}\times10^{13}\mathrm{K}$. At such a high temperature the EOS of electro-proton plasma is almost the same as that of the pure proton gas. The latter was used in our simulations.

Bowman’s solution is shown in the top part of Figure 1. As the external pressure decreases rapidly, the jet quickly becomes under-expanded and enters the phase of almost free expansion. When it enters the outer region of constant pressure it becomes over-expanded and a reconfinement shock is pushed towards its axis, where it gets reflected. Gas passed though these two shocks becomes hot and its pressure rises. As a result, the jet becomes somewhat under-expanded again and begins to expand for the second time. Then it becomes over-expanded again and another shock is pushed into the jet and so on.

In the bottom part of this figure, we show the results of our 1D simulations for this jet using exactly the same visualization technique as in the original paper. The agreement between the two solutions is quite remarkable. A very good match for the maximal radial extension and the oscillation-length of the jet is obtained. The successive reconfinement shocks are somewhat sharper than in B94, most likely due to the application of a shock-capturing scheme. We checked our approach against other numerical models of B94 as well. In all models, the results for profile of jet radius and Mach number are in good agreement. Noticeable but still minor differences arise only for the colder models, most likely due to the different equation of state used in our simulations.

### Self-similar models of jet reconfinement

The problem of reconfinement of initially free-expanding steady-state jets is quite important and a number of authors have tried to find simple analytic of semi-analytic solutions. Falle (1991) and Komissarov and Falle (1997) used the Kompaneets approximation, which assumes that the gas pressure immediately downstream of the reconfinement shock is equal to the external pressure at the same distance, to derive a simple ODE for the shock radius. Assuming particular flow profiles in the shocked layer, one can also determine the location of the jet boundary (*e.g.* Bromberg and Levinson 2007). The Kompaneets approximation is accurate only for very narrow jets. To improve on it, one also has to take into account the variation of the gas pressure across the shocked layer (Nalewajko and Sikora 2009). In our second test, we compare our results with the semi-analytical model by Kohler et al. (2012), thereafter KBB12, who assumed self-similarity of the flow in this layer. This assumption is more suitable for the case where the reconfinement shock never reaches the jet axis, because otherwise the distance where this occurs sets a characteristic length scale.

KBB12 studied jets with ultra-relativistic equation of state ($w=4p$, $\gamma=4/3$), propagating in a power-law atmosphere,

33 $$ p = p_{0} \biggl(\frac{z}{z_{0}} \biggr)^{-\kappa} . $$

These jets emerge from a nozzle at $z=z_{0}$ with the Lorentz factor $\Gamma_{0}$, opening angle $\theta_{0}=1/\Gamma_{0}$ and pressure $p_{0}$. The initial velocity distribution correspond to a conical flow originating from $z=0$ and hence the initial jet radius $r_{0}=z_{0}\tan(\theta_{0})$. They could only find self-consistent solutions for $8/3 \le\kappa<4$ and later argued that for $\kappa<8/3$ the entropy of the shocked layer must increase with the distance along the jet in order for the solution to be consistent with the energy conservation (Kohler and Begelman 2015). They proposed that this additional heating is caused by multiple shocks driven into the flow as it gradually collimates.

**Figure 2:**
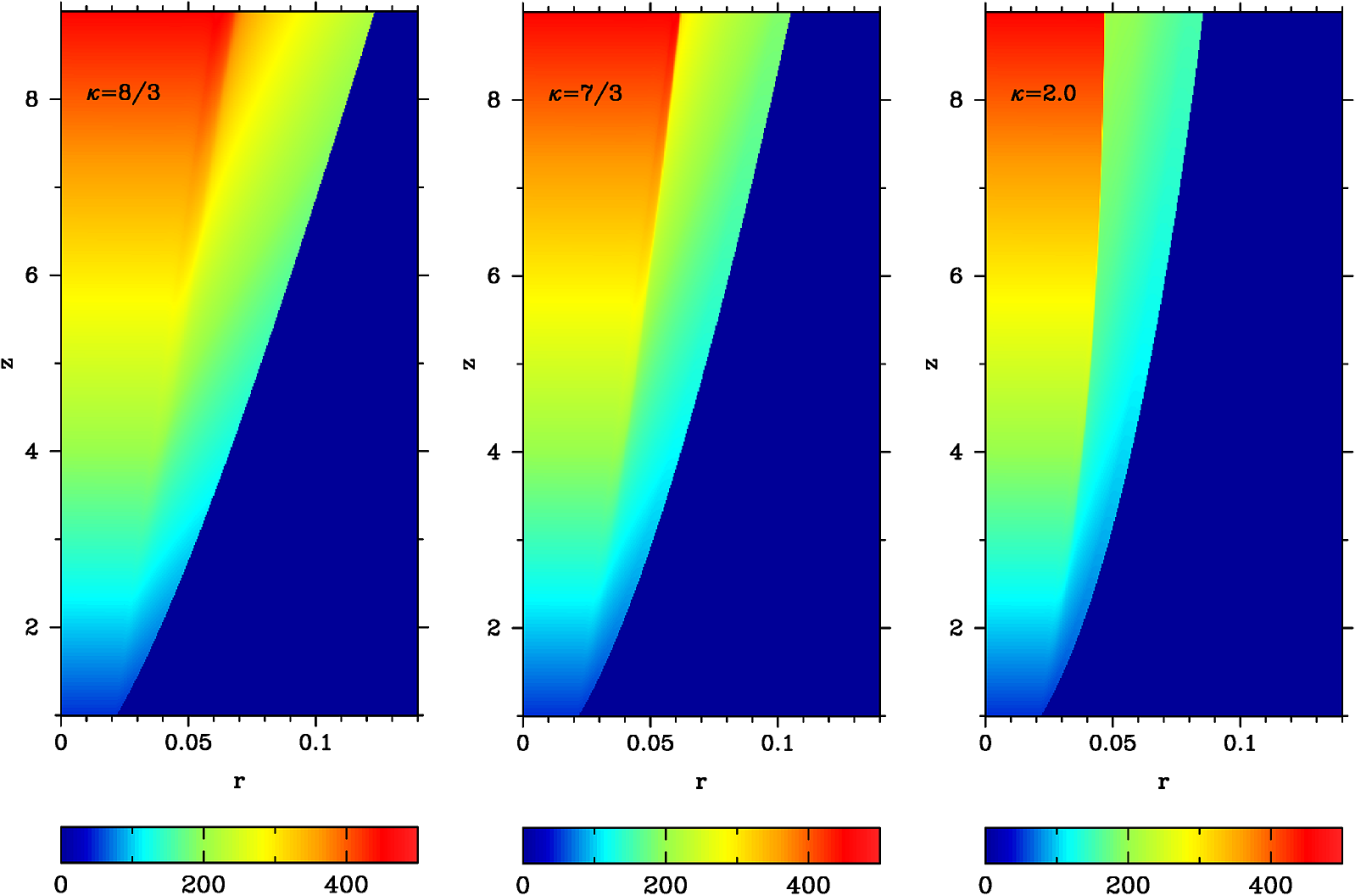
**Ultra-relativistically hot jets (Kohler et al.** 2012 **; Kohler and Begelman** 2015 **) in power-law atmospheres with** $\pmb{\kappa=8/3,7/3}$ **and 2 (from left to right).** The color-coded images show the distribution of the Lorentz factor. The initial Lorentz factor is $\Gamma_{0}=50$ and opening angle $\theta_{0}=1/\Gamma_{0}$.

We selected the KBB12 model with $\kappa=8/3$ and $\Gamma_{0}=50$ and made simulations on a uniform grid with only 300 cells (each run took only several CPU minutes on a laptop using only one core of its processor). Our results are shown in the first panel of Figure 2, which should be compared with Figure 7 in KBB12. Again we find a very good agreement between the models - at $z=9$ we have got the jet radius $r_{\mathrm {j}}\approx0.114$ and the shock radius $r_{\mathrm {s}}\approx0.07$, whereas in KBB12 $r_{\mathrm {j}}=0.110$ and $r_{\mathrm {s}}=0.064$.

**Figure 3:**
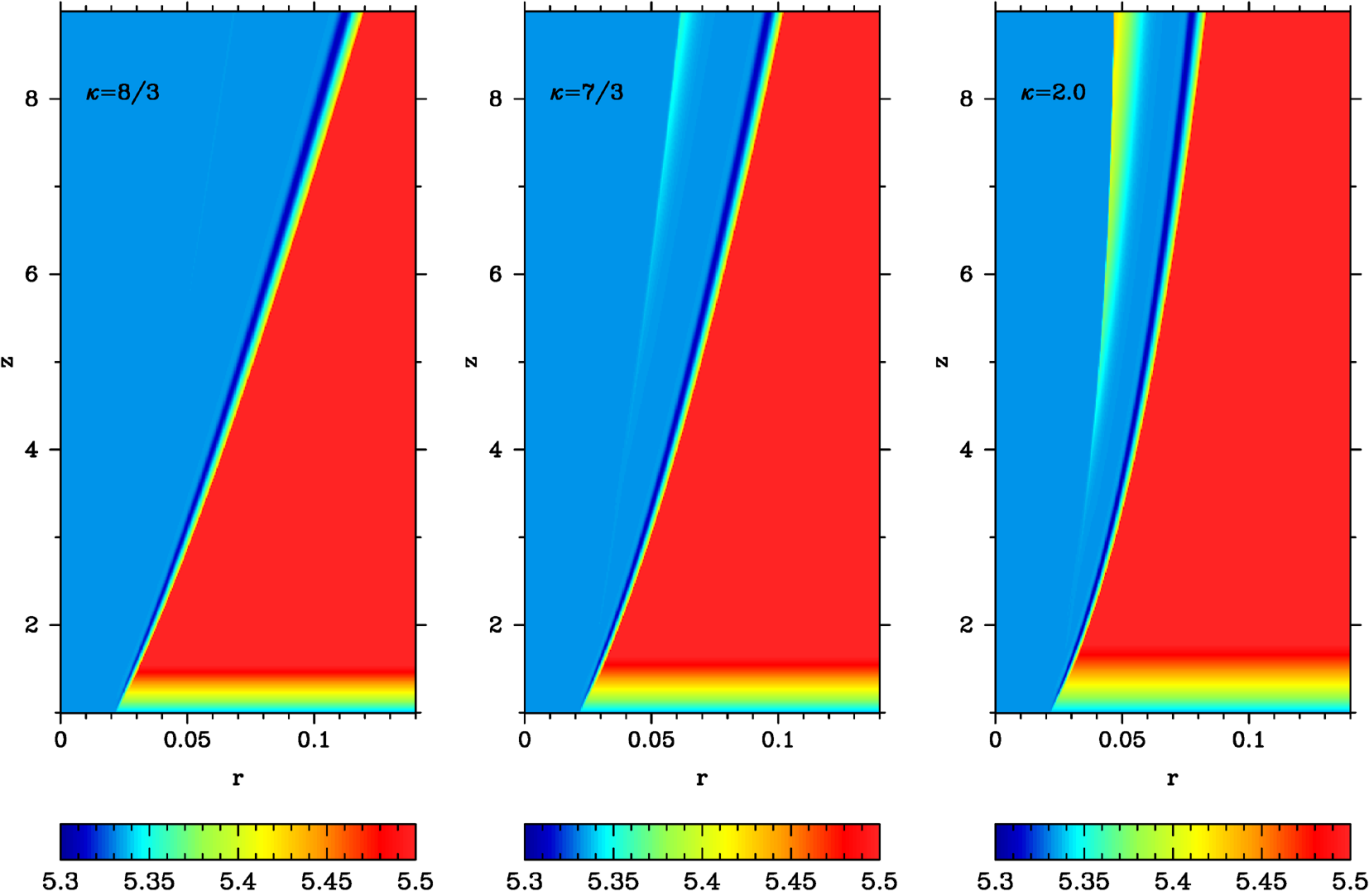
**Ultra-relativistically hot jets (Kohler et al.** 2012 **; Kohler and Begelman** 2015 **) in power-law atmospheres with** $\pmb{\kappa=8/3, 7/3}$ **, and 2 (from left to right).** The color-coded images show $\log_{10}(p\rho^{-\gamma})$. The dark blue region along the jet boundary is obviously a numerical artifact as its entropy is lower than that of the initial solution anywhere on the grid.

In order to understand the difference between the cases with $\kappa >8/3$ and $\kappa<8/3$, we also computed models with $\kappa=7/3$ and 2 - the evolution of the Lorentz factor in these models is shown in the second and third panels of Figure 2 respectively. In these plots we see no evidence of the additional shocks proposed in Kohler and Begelman (2015). Neither could we find them in plots of other parameters. However, Figure 2 suggests that in the models with $\kappa=7/3$ and 2 the reconfinement shock is much stronger than in the model with $\kappa=8/3$. Moreover, the shock strength is increasing with the distance along the jet. As the result, the entropy of the shocked layer in the models with $\kappa=7/3$ and 2 is higher and its mean value across the layer is growing with the distance. This is confirmed in Figure 3, which shows the entropy distribution for these models. Since KBB12 assumed isentropy of the flow in the shocked layer, this could be the reason why their self-similar model fails for $\kappa<8/3$. In contrast, in the model with $\kappa=8/3$ the mean entropy of the layer does remain fairly constant. Based on these results, we conclude that the value of $\kappa=8/3$ is not special, but the accuracy of the constant-entropy approximation used in KBB12 greatly reduces as *κ* decreases.

The plots in Figure 3 also reveal a thin layer of decreased entropy stretching along the jet boundary. As in this layer the entropy is lower than anywhere in the initial solution, this is definitely a numerical artifact. We have checked that it becomes less pronounced with increased numerical resolution. Moreover, this layer forms well inside the jet and thus its origin is not related to the resetting procedure but is a property of our time-dependent code.

**Figure 4:**
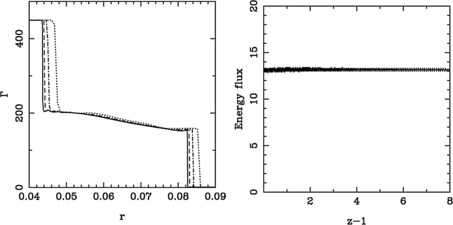
**Accuracy of the ultra-relativistic hot jets solution for the model with atmospheres with** $\pmb{\kappa=2}$ **.** The left panel shows the Lorentz factor at $z=9$ for models with 150 (dotted), 300 (dot-dashed), 600 (dashed line) and 1,200 (solid line) grid points. The right panel show the total energy flux as a function of the distance from the nozzle for the model with 300 grid points.

We choose the model with $\kappa=2$, to illustrate the convergence and accuracy of our numerical solutions. The left panel of Figure 4 shows the Lorentz factor distributions found at $z=9$ for runs with different number of grid cells in the computational domain, increasing from 150 to 1,200 cells. As one can see, the solutions converge as in a first-order accurate scheme. The right panel shows the evolution of the total energy flux along the jet. It remains fairly constant, as expected for a conserved quantity. As the jet boundary jumps from one cell to another, a low level noise is introduced to this integral variable.

### Magnetized jets. 1D versus 2D solutions

The steady-state structure of magnetized jets is more complex, mainly due to the non-trivial contribution of the magnetic tension to the force balance. A number of authors have tackled this problem analytically using various approximations (*e.g.* Zakamska et al. 2008; Lyubarsky 2009; Lyubarsky 2010; Kohler and Begelman 2012). However, none of these studies deliver a model suitable for detailed testing of our numerical approach. Dubal and Pantano (1993) studied the steady-state structure of relativistic jets with azimuthal magnetic field using the method of characteristics. This would be a good test case but the setup of their simulations is ambiguous. We have tried several variants of the setup but each time failed to reproduce the results. The mechanisms of magnetic collimation and acceleration of relativistic jets were studied numerically by Komissarov et al. (2007), Komissarov et al. (2009) and Tchekhovskoy et al. (2010) using a ‘rigid wall’ outer boundary. While this allows for a well-controlled experiment, Komissarov *et al.* (2009) have shown that the connection between the shape of the boundary and the external pressure gradient is not straightforward, with significant degeneracy. For this reason, we concluded that in the magnetic case the best way of testing the performance of our 1D approach would be via new 2D axisymmetric time-dependent simulations using the relativistic AMRVAC code (Keppens et al. 2012; Porth et al. 2014).

The problem we selected for this test is similar in its setup to the one described in Section 4.2 as it also involves a jet propagating through the atmosphere with the power-law pressure distribution (33), and the nozzle is still located at $z=z_{0}$. However, this time the jet is magnetized and the rest mass density of its particles is not negligibly small. The jet structure at the inlet is that of a cylindrical jet in magnetostatic equilibrium, which satisfies the following force balance equation

34 $$ \frac{d p_{t}}{d r} + \frac{b^{\phi}}{r} \frac{d rb^{\phi}}{d r} =0 , $$

where $b^{\phi}=B^{\phi}/\Gamma$ is the azimuthal component of the magnetic field as measured in the fluid frame using normalized basis and $p_{t}$ is the sum of the gas pressure and the magnetic pressure due to the axial magnetic field $B_{z}$ (Komissarov 1999). Equation (34) has infinitely many solutions - given a particular distribution for $b^{\phi}(r)$ one can solve this equation for the corresponding distribution of the pressure $p_{t}(r)$. We adopted the ‘core-envelope’ solution of Komissarov (1999):

35 $$\begin{aligned}& b^{\phi}(r) = \left \{ \textstyle\begin{array}{@{}l@{\quad}l} b_{m}(r/r_{m}) ;& r< r_{m}, \\ b_{m}(r_{m}/r) ;& r_{m}< r< r_{j},\\ 0;& r>r_{j}, \end{array}\displaystyle \right . \end{aligned}$$

36 $$\begin{aligned}& p_{t}(r) = \left \{ \textstyle\begin{array}{@{}l@{\quad}l} p_{0} [\alpha+\frac{2}{\beta_{m}}(1-(r/r_{m})^{2}) ] ;& r< r_{m},\\ \alpha p_{0} ;& r_{m}< r< r_{j}, \\ p_{0} ;& r>r_{j}, \end{array}\displaystyle \right . \end{aligned}$$

where

37 $$ \beta_{m}= \frac{2 p_{0}}{b_{m}^{2}},\qquad \alpha=1-(1/\beta _{m}) (r_{m}/r_{j})^{2} , $$

**Figure 5:**
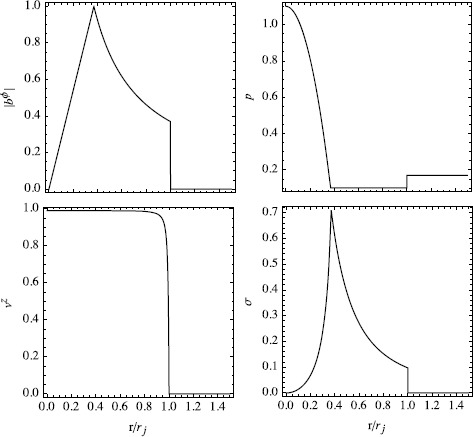
**Initial radial structure of the magnetized jets in the test simulations described in Section** **4.3** **.**

$r_{j}$ is the jet radius and $r_{m}$ is the radius of its core (Note a typo in the expression for *α* in Komissarov (1999).). As one can see, the core is pinched and in the envelope the magnetic field is force-free. This may be combined with any distribution of density and axial velocity. We imposed $\rho =\rho_{0}$ and

38 $$ \Gamma(r) = \Gamma_{0} \bigl(1-(r/r_{j})^{\nu}\bigr) + (r/r_{j})^{\nu}, $$

with $\nu=8$; this gives an almost ‘top-hat’ velocity profile. The velocity vector is set to be aligned with the jet axis, so $v_{r}=v_{\phi}=0$. This solution is illustrated in Figure 5.

We considered two models, A and B. In the models A, the magnetic field is purely azimuthal and the other parameters are $r_{j}=1$, $r_{m}=0.37$, $b_{m}=1$, $\rho_{0}=1$, $z_{0}=1$, $\beta_{m}=0.34$, $\Gamma_{0}=10$. The local magnetization parameter $\sigma=b^{2}/w$ does not exceed $\sigma_{\max}=0.7$ in this model and thus the jet is only moderately magnetized. The jet core is relativistically hot, with the gas pressure reaching $p_{\max}=\rho$ at the axis, which opens the possibility of efficient hydrodynamic acceleration once the jet is allowed to expand. In the simulations we used the adiabatic equation of state $w=\rho+(\gamma/\gamma-1)p$ with $\gamma=4/3$.

In model B, this configuration is modified to include nonvanishing longitudinal magnetic field $B_{z}$. In particular, we considered the case where the gas pressure $p=\alpha p_{0}$ everywhere within the jet and

39 $$ B^{z} = \left \{ \textstyle\begin{array}{@{}l@{\quad}l} p_{0} [\frac{2}{\beta_{m}}(1-(r/r_{m})^{2}) ] ;& r< r_{m},\\ 0 ;& r>r_{m} , \end{array}\displaystyle \right . $$

which keeps $p_{t}$ unchanged. In this model, the magnetic field is force-free not only in the envelope but also in the core. The other parameters of this model that differ from those of model A are $\rho_{0}=0.05$ and $\beta_{m}=0.14$. The corresponding magnetization is much higher, with $\sigma_{\max}=17$.

Model B turned out too stiff for our 2D code, but presented no problems in 1D simulations. For this reason we compare here the 1D and 2D results for model A only. In these simulations we used the atmosphere with $\kappa=1$. The computational domain is $20 r_{j}$ in the radial direction and $800 r_{j}$ in the axial direction.

**Figure 6:**
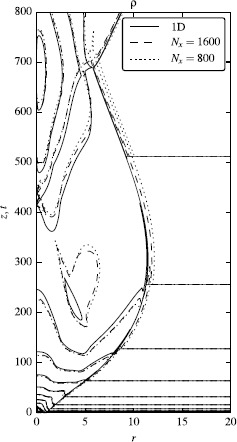
**Converged 1D solution for a stationary magnetized jet (solid lines) and two corresponding 2D solutions found via the relaxation method, one with the** $\pmb{1{,}600\times3{,}200}$ **-resolution (dashed lines) and one with the** $\pmb{800\times1{,}600}$ **-resolution (dotted lines).** The lines are 10 rest-frame density contours consecutively spaced by the factor of one half from the starting value of $\rho_{\max}=1$. The solution involves a reconfinement shock which reaches the jet axis at $z\approx400$.

First, let us describe the overall jet structure found in these simulations. Initially, as the jet enters the region of rapidly declining external pressure, it expands rapidly and a rarefaction wave moves towards its axis. Eventually, the jet becomes over-expanded, its expansion slows down, and a reconfinement shock sets in. It reaches the axis at $z\approx400$, gets reflected and then returns to the jet boundary at $z\approx700$ (see Figure 6).

To quantify the convergence of the 1D simulations we carried out simulations with three different resolution and used this data to determine the grid-convergence index

40 $$ \eta\equiv-\ln \biggl(\frac{|f_{2}-f_{1}|_{1}}{|f_{1}-f_{0}|_{1}} \biggr) /\ln{2}, $$

where $f_{1}$, $f_{2}$ are solutions with doubled and quadrupled resolution compared to $f_{0}$ and $|f_{a}-f_{b}|_{1}$ is the difference between two solutions in the $L_{1}$ norm. We found that $\eta\approx1$, as this is expected for a TVD scheme in the presence of discontinuities. At $6{,}400$ grid cells in the radial direction, the density contours become visually unchanged on the scale of Figure 6. The 1D solution with this resolution was used for comparison with the results of our 2D simulations. In what follows we refer to it as the ‘converged’ 1D solution.

The initial solution in our 2D simulations was constructed via interpolation of the converged 1D solution onto the 2D cylindrical grid. Since we did not include gravity to balance the pressure gradient in the external atmosphere, in order to preserve the atmosphere in its initial state the atmospheric parameters were reset to their initial values every time step, just like this was done in the 1D case. In order to test the convergence of 2D solutions, we made three runs with doubled resolution, $N_{r}=400, 800, \mbox{and }1{,}600$ cells in the radial direction. The number of cell in the axial direction was always twice the number of cells in the radial one.

Typically, the 2D solutions exhibited some evolution at first but then quickly settled into a stationary state. For example in the case of $N_{r}=400$, the timestep-to-timestep relative variation of the conserved flow variables dropped below $6\times10^{-6}$ at $t=1{,}000$ and remained approximately constant thereafter. Furthermore, the relative $L_{1}$ error of density between times $t=1{,}000$ and $t=3{,}000$ was $2.8\times10^{-4}$, indicating that a stationary state had been reached. The 2D solutions converge with the grid-convergence index $\eta>1.25$ over the entire simulated time.

The difference between the converged 1D solution and the relaxed 2D solutions with $N_{r}=800$ (dotted lines) and $N_{r}=1{,}600$ (dashed lines) is illustrated in Figure 6 which shows the mass density distribution. One can see that the 2D solutions are very close to the 1D solution and that the difference decreases with the resolution of 2D runs. To quantify the difference between the relaxed 2D solutions and the converged 1D approximate solution we introduce the parameter

41 $$ \delta\rho=|\rho_{2D}-\rho_{1D}|_{1}/\langle \rho_{1D}\rangle . $$

For the 2D solution with $N_{r}=400$ cells in the radial direction we obtain $\delta\rho\simeq6\%$, for $N_{r}=800$, $\delta\rho\simeq 4.3\%$ and for $N_{r}=1{,}600$ the relative error decreased to $\delta\rho\simeq 3.2\%$. This shows that the approximation error of our 1D approach is at the level of no more than 3%.

### Magnetized jets in power-law atmospheres

Komissarov et al. (2009) derived an approximate equation for the radius of highly magnetized jets, in the limit where it strongly exceeds that of the light cylinder. Using this equation they concluded that in the case of power-law atmosphere with $0<\kappa<2$ the jet radius increases as

42 $$ r_{j} \propto z^{\kappa/4} . $$

Lyubarsky (2009) developed the theory of Poynting-dominated jets further and using more accurate analysis found that the expansion is modulated by oscillations with the wavelength growing as

43 $$ \lambda\propto z^{\kappa/2}. $$

These oscillations can be understood as a standing magneto-sonic wave bouncing across the jet. Indeed, denote the wave speed as $a_{m}$. Then the jet crossing time is $\tau_{c}=r_{j}/a_{m}$ in the co-moving jet frame and $t_{c}=\Gamma\tau_{c}$ in the rest frame of the atmosphere. As the wave is advected along the jet almost at the speed of light the wavelength of the associated structure is

44 $$ \lambda\simeq\Gamma\frac{c}{a_{m}} r_{j} . $$

Since for the jets considered in Komissarov et al. (2009) and Lyubarsky (2009) $a_{m}\simeq c$ and $\Gamma\propto r_{j}$ we obtain $\lambda\propto r_{j}^{2}$ and using Eq. (42) recover Eq. (43). The results (42,43) are well suited for testing of our approach. To this aim, we carried out additional 1D simulations with models A and B described in Section 4.3.

Since in model A the jet is not Poynting-dominated, it allows us to explore the regime not covered in Lyubarsky (2009). To see how sensitive these results may be to the assumptions made in Komissarov et al. (2009) and Lyubarsky (2009) let us consider unmagnetized relativistic jets. From the mass conservation law we obtain $r_{j} \propto(\Gamma\rho)^{-2}$. For relativistically cold jets with $p\ll\rho c^{2}$ we have $\Gamma\simeq\mbox{const}$ and thus

45 $$ r_{j} \propto z^{\kappa/2\gamma} , $$

whereas for the hot jets $\Gamma\propto r_{j}$ and thus

46 $$ r_{j} \propto z^{\kappa/4} , $$

where we put $\gamma=4/3$. The last result is the same as for the Poynting-dominated jets. Even for the cold jets the difference is rather minor, *e.g.* for $\gamma=5/3$ the index in Eq. (45) differs from $\kappa/4$ only by $\kappa/20$ and for $\gamma=4/3$ by $\kappa/8$.

In order find *λ* we note that for cold jets $a_{m}^{2} \propto(p/\rho) \propto z^{-\kappa(1-1/\gamma)}$ and hence Eq. (44) yields Eq. (43) independently of the value of *γ*. For hot jets, $a_{m} \simeq\mbox{const}$ and Eq. (44) still leads to Eq. (43) if we use $\gamma=4/3$. Thus, the law (43) for the wavelength of oscillations is very robust.

**Figure 7:**
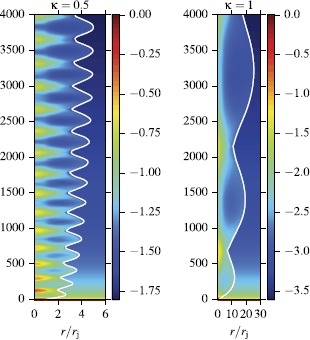
**Structure of steady-state magnetized jets obtained via time-dependent 1D simulations.** The plots show the co-moving density distribution for model A with $\kappa=0.5$ and $\kappa=1$. The distance along the vertical axis is defined as $z=ct/r_{j}$, where $r_{j}$ is the initial jet radius. The white contour shows the jet boundary, located using the passive scalar.

Figure 7 illustrates the overall jet structure in model A and its response to changes in the parameter *κ* of the external atmosphere. One can see that this weakly magnetized jet also shows a combination of secular expansion and oscillations. These oscillations appear to be a generic feature of the adjustment process of supersonic jets to variations of external pressure, which occurs by means of magneto-sonic waves traveling across the jet. In the very beginning, the decrease of external pressure makes the jet under-expanded and a rarefaction wave is launched from the jet boundary towards the jet axis. Behind this wave the radial velocity is positive and the flow expands. The rarefaction reduces the jet pressure and at some point it becomes over-expanded. Now a compression wave is driven inside the jet. Behind this wave the jet expansion slows down and eventually turns into a contraction. The contraction increases the jet pressure and at some point it becomes under-expanded again and then the whole cycle repeats.

The deviation from the force-balance corresponding to the secular jet expansion is due to the finite propagation speed of the waves - as they move across the jet they are also advected downstream by the supersonic flow. As the result, the jet interior reacts to the changes in the external pressure with a delay. It keeps expanding when the internal pressure is already too low and keeps contracting when it is already too high. As *κ* increases, the wavelength of the oscillation increases as well. This is expected as the more rapid overall expansion of the jet in an atmosphere with larger *κ* means that it takes longer for a magneto-sonic wave to traverse the jet, not only as the result of the larger jet radius but also as the result of its higher Mach number (and hence smaller Mach angle).

Overall, this is very similar to the well-known evolution of under-expanded supersonic jets studied in laboratories. Normally, their compressive transverse waves steepen into shocks. In our model A with $\kappa=1$ we also detect shocks, but they become progressively weaker, suggesting that they may disappear further out along the jet. For $\kappa=0.5$, shocks do not form at all. The exact reason for this in not yet clear.

**Figure 8:**
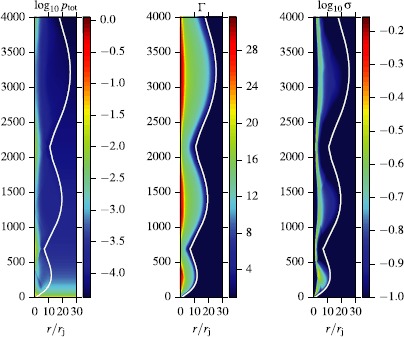
**Structure of the steady-state magnetized jet in model A with** $\pmb{\kappa=1}$ **, obtained via time-dependent 1D simulations.** From left to right, the plots show the total pressure, Lorentz factor and the magnetization parameter *σ*. Jet oscillations cause compression in the squeezed regions as well as re-acceleration of the bulk flow as the flow expands. The majority of acceleration occurs in the thermally dominated core. A reconfinement shock is clearly visible in the total pressure and magnetization plots.

**Figure 9:**
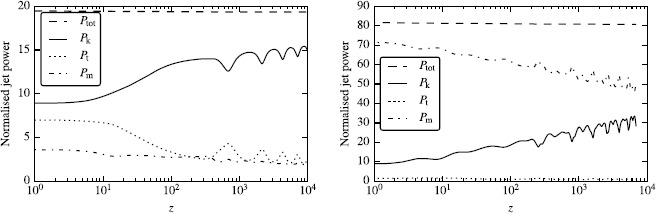
**Evolution of energy fluxes with the distance along the jet in models A (left panel) and B (right panel).** The curves show fluxes of the total (dash), kinetic (solid), thermal (dot) and magnetic (dot-dash) energies. Each is normalized to the rest-mass flux.

Figure 8 shows the evolution of other flow parameters in model A with $\kappa=1$. Both the secular and oscillatory behavior of the jet radius are mirrored in the variation of the Lorentz factor. The secular expansion leads to secular increase of the Lorentz factor as both the thermal and the magnetic energy are converted into the kinetic energy of the flow. The thermal acceleration is most pronounced in the jet core, which is relativistically hot at the inlet. The oscillations of the jet radius lead to additional increase of the Lorentz factor during the expansion phase and its decrease upon contraction. The left panel of Figure 9 shows the dynamics of energy fluxes for this jet. These are found via integration over the jet cross-section of $b^{2} \Gamma^{2} v^{z} -b^{0}b^{z} $ for the magnetic energy, $\rho\Gamma ^{2} v^{z}$ for the kinetic energy and $(w-\rho)\Gamma^{2} v^{z}$ for the thermal energy. The results are normalized to the rest-mass flux, obtained via integration of $\rho\Gamma v^{z}$. As the result of this normalization, the kinetic energy flux has the meaning of mean actual Lorentz factor of the jet, whereas for the thermal energy and magnetic energies these are the gains in the Lorentz factor, which can be achieved upon full conversion of these energies into the kinetic one. The main feature of the plot is a conversion of the thermal energy into the kinetic one (the magnetic energy is highly sub-dominant from the start). This conversion is largely completed during the initial phase of monotonic expansion, which lasts up to $z=200$. In the second phase, the thermal energy flux is comparable to the magnetic, and they are being converted to the kinetic energy at more of less the same and rather slow rate. Strong oscillations are superimposed upon this secular evolution, with the kinetic (thermal) energy reaching local maxima (minima) at the locations of jet bulging.

**Figure 10:**
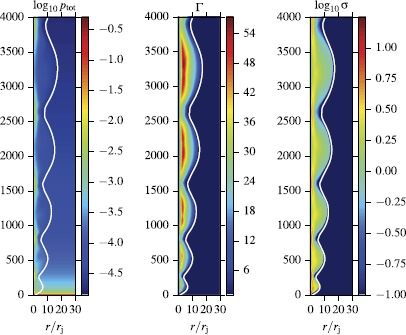
**As Figure** **8** **for model B with** $\pmb{\kappa=1}$ **.** As the Poynting-flux vanishes on the axis (and the thermal energy is negligible), we obtain a hollow jet with fastest region away from the axis. Due to the increased fast-magneto-sonic speed (thus lower Mach-number) compared to the case of Figure 8, no reconfinement-shock forms and the jet-oscillation frequency is increased.

Figure 10 shows the same parameters for the highly magnetized jet of model B with $\kappa=1$. In this model, the reconfinement shock is no longer present. This may be related to the fact that in this model the fast magneto-sonic speed is higher and the corresponding jet Mach number is lower, at the inlet $M\simeq3$ compared to $M\simeq10$ in model A. The lower Mach number is also responsible for the observed lower wavelength of the jet oscillations as it takes less time for the waves to traverse the jet. In this model, the jet is magnetically-dominated and the main feature of its energy balance is a gradual conversion of the magnetic energy into the kinetic one (see the right panel of Figure 9).

**Figure 11:**
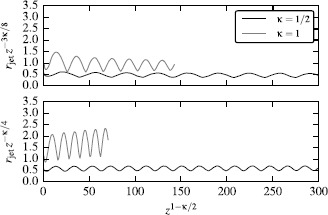
**Compensated jet-expansion laws for models A (top) and B (bottom).** In both models the expected average expansion is captured quite well. To show that the oscillation wavelength scales as $\lambda\propto z^{\kappa/2}$, the x-axis has been rescaled accordingly. In order to visually separate the curves corresponding to different values of *κ*, they have been shifted up by a factor of *κ*.

The theoretical predictions for the secular evolution of the jet radius and the wavelength of its oscillations are put to a quantitative test in Figure 11, which shows the jet radius rescaled according to its expected secular evolution against $z^{1-\kappa/2}$. In such plots, the mean jet radius and the wavelength of oscillations should remain constant. For the highly magnetized jet of model B the scaling factor is $z^{\kappa/4}$ and for the low magnetized jet of model A it is $z^{3\kappa/8}$, as appropriate for a cold hydrodynamic jet with $\gamma=4/3$. In general, we obtain a very good agreement with the theoretical scalings for the mean jet radius, both in the low- and high-magnetization limit. A small departure from the $z^{\kappa/4}$-scaling is observed for case B with $\kappa=1$ - it expands slightly faster. This could be because the jet magnetization is not sufficiently high and decreases more rapidly with distance than in the atmosphere with $\kappa=0.5$. The evolution of the wavelength scaling is also in a very good agreement with the theory - the residual error is between 0.7% and 3.4%.

## Conclusions

In this paper we presented a novel numerical approach, which can be used to determine the structure of steady-state relativistic jets. It is based on the similarity between the two-dimensional steady-state equations and the one-dimensional time-dependent equations of SRMHD with the cylindrical symmetry in problems involving narrow highly-relativistic ($v_{z}\approx c$) flows. Such similarity has already been utilised in the so-called ‘frozen pulse’ approximation where dynamics of time-dependent relativistic flows is analyzed using the steady-state equations (Piran et al. 1993; Vlahakis and Königl 2003; Sapountzis and Vlahakis 2013). Here we do the opposite and construct approximate steady-state solutions via numerical integration of the time-dependent equations. The main advantage of this approach is utilitarian. First, it allows us to use computer codes for relativistic MHD (or hydrodynamics in the case of unmagnetized flows), which are now widely available, in place of highly-specialised codes for integrating steady-state equations, which are not openly available at the moment. Moreover, the reduced dimensionality means that the computational facilities can be very modest - a basic laptop will suffice. In contrast, the relaxation method based on integration of two-dimensional time-dependent equations can be computationally quite expensive.

We compared numerical solutions obtained with this approach with analytical models and numerical solutions obtained with other techniques. The considered problems involved a variety of flows both magnetized and unmagnetized, with different equations of state and external conditions. The results show that the method is sufficiently accurate and robust.

Although we focused only on relativistic flows, we see no reason why this approach cannot be applied to non-relativistic hypersonic flows. For such flows, the axial velocity of bulk motion plays the role of the speed of light in the substitution $z=ct$ used in our derivations.

As a byproduct of our test simulations, we obtained two results of astrophysical interest. We demonstrated that the failure of the self-similar model of the jet reconfinement in power-law atmospheres with the index $\kappa<8/3$ (Kohler et al. 2012) is rooted in the assumption of isentropy of the shocked layer, which is made in this model. In reality, the reconfinement shock becomes stronger with the distance along the jet, resulting in a strong spatial variation of the entropy. We also found that the radial oscillations of steady-state jets, discovered in the analytical models of Poynting-dominated jets (Lyubarsky 2009) is a generic part of the jet adjustment to the space-variable external pressure and not specific to the high-magnetization regime only. The oscillations are standing waves induced by the variation.

The steady-state solutions are useful for elucidating some key factors in flow dynamics and may closely describe some of the observed phenomena in astrophysical jets. However, they are often subject to various instabilities which may dramatically modify the flow properties. Most instability studies, both analytical and numerical, deal with very simple problems where the steady-state solution is readily available. In more realistic setup, the issue of finding the steady-state solution, which can then be subjected to perturbations, becomes more involved and this is where our method can be applied in the instability studies.
